# Optimising patient recall of adverse events over prolonged time periods

**DOI:** 10.1186/1745-6215-12-S1-A74

**Published:** 2011-12-13

**Authors:** Louise Hiller, Janet A Dunn, Helen B Higgins, Emma Ogburn-Storey, Shrushma Loi, Anne-Laure Vallier, Helena M Earl

**Affiliations:** 1 Warwick Clinical Trials Unit, University of Warwick, Coventry, West Midlands, CV4 7AL, UK; 2 Cambridge Cancer Trials Centre, Addenbrooke’s Hospital, University of Cambridge, CB2 0QQ, UK; 3 Department of Oncology, Addenbrooke’s Hospital, University of Cambridge, CB2 0QQ, UK

## Objectives

Large, simple, pragmatic, low-risk, maintenance treatment trials may seem straightforward to design, run and analyse. However, if trial case record forms (CRFs) are completed infrequently, the reliability of patient recall over these long time-periods is questionable, especially when recording individual incidences and severity of toxicities of specific interest to the trial. It is essential to know these detailed toxicities as it forms an indication of the reduced level of exposure when comparing treatment duration in a non-inferiority trial. Optimal methods of achieving accurate data are required.

## Methods

PERSEPHONE is an investigator-led phase III trial of trastuzumab treatment duration where patients on each trial arm are treated every 21 days for either 9 or 18 cycles of treatment. Treatments are administered in chemotherapy outpatient departments or at the patient’s home via Healthcare at Home Ltd. PERSEPHONE CRFs, which include all adverse events of interest, are completed by the site research team at the 3-monthly clinic visits where PERSEPHONE patients are routinely followed up for the first year following start of trastuzumab treatment.

To optimise reporting, the PERSEPHONE team have developed Patient Diary Sheets which list the common expected toxicities. For each trastuzumab cycle, patients are requested to record how much they are ‘troubled by each of the possible side-effects’ using patient-friendly severity ratings (0=Not at all, 1=A little, 2=Moderate, 3=Quite a bit, 4=Very much).

## Results

To date 1260 patients have been recruited into the PERSEPHONE trial with adverse event information summarised on over 10,000 trastuzumab doses. At the 3-monthly clinic visits, the site research team discuss the detailed patient diary information with the patient and accurately interpret the information into incidences of toxicities and relevant Common Terminology Criteria for Adverse Events (CTCAE) grades for entry onto the trial CRFs. The patient diaries are not entered onto the database for analysis as they are used solely as memory aids.

## Conclusions

Feedback from the clinical research teams at sites indicates that the method adopted in the PERSEPHONE trial appears to work well. The Patient Diary Sheet is indeed well received and utilised by the patients and reported as a very useful memory aid at clinic visits. Whilst it may seem counterintuitive to ask for additional data which will never be scrutinised by the PERSEPHONE trial team, the strength of the final data collected has been optimised by the use of these data triggers.

